# High pretreatment serum gamma-glutamyl transpeptidase predicts an inferior outcome in nasopharyngeal carcinoma

**DOI:** 10.18632/oncotarget.18798

**Published:** 2017-06-28

**Authors:** Min Luo, Wei Sun, Cheng Wu, Linli Zhang, Dongbo Liu, Wenwen Li, Qi Mei, Guoqing Hu

**Affiliations:** ^1^ Department of Oncology, Tongji Hospital, Tongji Medical College, Huazhong University of Science and Technology, Hubei, Wuhan, People's Republic of China

**Keywords:** nasopharyngeal carcinoma, serum marker, gamma-glutamyl transpeptidase, survival, prognosis

## Abstract

**Background:**

Gamma-glutamyl transpeptidase (GGT) which plays an important role in tumor initiation, invasion, drug resistance is strongly associated with poor prognosis in patients with cancers. This study was designed to estimate whether pretreatment serum GGT could predict the clinical outcome of nasopharyngeal carcinoma (NPC) patients.

**Results:**

An optimal cutoff value was identified as 23 U/L for GGT. Univariate analysis and multivariate analysis demonstrated that elevated GGT was correlated with shorter local recurrence-free survival (LRFS) (HR, 4.163; 95% CI, 1.690-10.251; p=0.023), progression-free survival (PFS) (HR, 3.119; 95% CI, 1.955-4.976; p=0.031) and overall survival (OS) (HR, 2.811; 95% CI, 1.614-4.896; p=0.007).

**Materials and Methods:**

We retrospectively analyzed data from 374 patients with NPC. Kaplan–Meier method was used to calculate and compare the prognosis. The Cox proportional hazards model was applied to carry out univariate and multivariate analyses.

**Conclusion:**

Pretreatment GGT can be a novel and independent prognostic biomarker for patients with NPC.

## INTRODUCTION

Nasopharyngeal carcinoma (NPC) is one of the most common head and neck cancers in Southeast Asia, especially in Southern China. With the rapid development of radiotherapy technology and chemotherapy regimens, the 5-year survival rate of NPC has reached approximately 80% [1, 2]. However, locoregional recurrence and distant metastasis after treatment still remain the main failure patterns affecting the survival rate of patients with advanced NPC [3]. Thus, searching for biomarkers associated with the prognosis of NPC patients are urgently needed [4]. In recent years, plenty of evidences had shown that some molecular makers, such as lactate dehydrogenase (LDH) [5], neutrophil-lymphocyte ratio, platelet-lymphocyte ratio [6], Epstein-Barr virus [7] and c-reactive protein [8] could successfully stratify patients regarding to their prognosis. Tumor-nodes-metastasis (TNM) classification is currently the gold standard for risky group classification. However, patients even with the same TNM stages could have dramatically different survival results [7]. These inexpensive, objective and easily detected markers are of great value to complement the TNM staging system.

Gamma-glutamyl transpeptidase (GGT) is a membrane-bound enzyme which can modulate the metabolism of glutathione (GSH) [9]. Located on the outer aspect of the cell membrane, GGT catalyzes the degradation of extracellular GSH and cleaves extracellular glutathione. By this way, GGT can provide the cells with access to additional cysteine and contribute to cellular antioxidant/antitoxic defenses [10]. Meanwhile, GGT may play a role in tumor initiation, invasion and drug resistance which could also predict poor prognosis of patients with cancers [11–13]. GGT included in routine biochemical examination was also showed as a promising prognostic factor for various cancers [14–20]. As a result, we supposed that GGT, a serum enzyme which can be acquired easily, may be of great value to predict prognosis in patients with NPC and guide individual treatment.

However, little is known about the association of pretreatment GGT and prognosis in patients with NPC. Therefore, we performed the present study to evaluate the association between pretreatment GGT and clinical outcome in patients with NPC.

## RESULTS

### Patient characteristics

In the end of follow-up, 374 patients were included and 145 patients were excluded. More details about how to select the patients into the study team were shown in Supplementary Table 1. The median age of diagnosis was 46 years. 263 were male and 111 were female, with a sex ratio of 2.4:1. There were 92 and 282 patients with stage I+II and III +IV disease, respectively. Chemotherapy was administered to 370 patients, while the other four was given radiotherapy alone. Concurrent chemoradiotherapy (CCT) was delivered to 66 patients, neoadjuvant chemotherapy (NACT) + CCT to 228 patients, CCT + adjuvant chemotherapy (ACT) to 6 patients, NACT + CCT + ACT to 74 patients. During follow-up, 92 patients (24.6%) experienced tumor progression after treatment. By the end of follow-up, 65/374 patients (17.4%) had died. The 5-year local recurrence-free survival (LRFS), distant metastasis-free survival (DMFS), progression-free survival (PFS) and overall survival (OS) rates were 92.2%, 83.5%, 75.4% and 82.6%, respectively.

### ROC analysis

**Figure 1:**
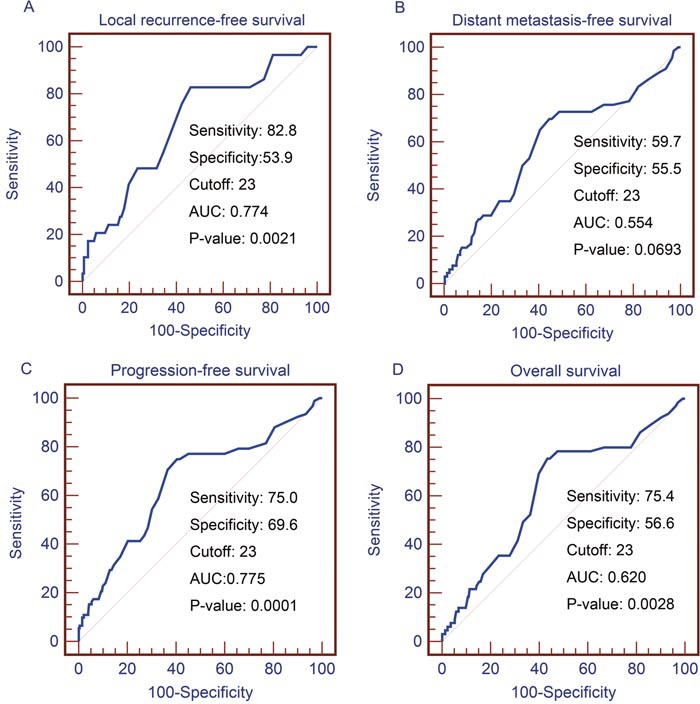
ROC analyses for the pretreatment serum GGT level **(A)** Using LRFS as an end point, the cut-off values provided by ROC analysis were 23 for GGT. Using DMFS **(B)**, PFS **(C)** and OS **(D)** as an end point, the cut-off values were the same. ROC, receiver-operating characteristic; GGT, gamma-glutamyl transpeptidase; LRFS, local relapse-free survival; DMFS, distant metastasis-free survival; PFS, progression-free survival; OS, overall survival.

Best cutoff value was determined by Receiver-operating characteristic (ROC) curve generated from MedCalc, with the highest combined sensitivity and specificity respecting to 5-year LRFS, 5-year DMFS, 5-year PFS and 5-year OS. Using 5-year LRFS as an end point, the cut-off value provided by ROC analysis was 23U/L for GGT. As showed in Figure 1A, area under the curve (AUC) of GGT is 0.774 (p=0.0021). Therefore, GGT could be identified as a potential prognostic biomarker in LRFS analysis. However, positive result was not acquired for DMFS, its AUC was 0.554 and p value was 0.0693. The ROC curve for DMFS was presented in Figure 1B. GGT could be used as a prognostic biomarker for PFS and OS analysis with an optimal AUC (0.755, 0.620, respectively) and p value (0.0001, 0.0028, respectively), which was presented in Figure 1C and Figure 1D. These values calculated by ROC analysis were adopted in subsequent survival analysis and used to stratify patients into different groups.

### Association between elevated GGT (≥23 U/L) and clinical characteristics of patients with NPC

Of the 374 patients, Elevated pretreatment GGT defined as ≥23 U/L based on our scan of cut-off values described above was detected in 185 NPC patients (49.5%). Patients classified as male (p<0.001), N2+3 (p=0.013) and higher TNM stage (p=0.031) were more likely to have elevated GGT (Table 1). In contrast, the frequency of elevated GGT was similar between the following subgroups of patients: those classified as WHO Type2 and WHO Type 3 (p=0.758), T1+2 and T3+4 (p=0.605).

**Table 1:** Associations of serum level of GGT and clinical–pathological characteristics in NPC

Parameter	Pretreatment serum GGT level n(c%)		P-value
	<23 U/L	≥23U/L	
**Gender**			
Female	79(41.8)	32(17.3)	
Male	110(58.2)	153(82.7)	<0.001
**Histology**			
WHO Type 2	94(51.4)	95(49.7)	
WHO Type 3	89(48.6)	96(50.3)	0.758
**Age at diagnosis (years)**			
<50	133(51.2)	60(52.6)	
≥50	127(48.8)	54(47.4)	0.473
**Tumor classification**			
T1+2	98(51.9)	90(48.6)	
T3+4	91(48.1)	95(51.4)	0.605
**Lymph node classification**			
N0+1	102(54.0)	76(41.1)	
N2+3	87(46.0)	109(58.9)	0.013
**TNM stage(AJCC)**			
I+II	55(29.1)	36(19.5)	
III+IV	134(70.9)	149(80.5)	0.031

GGT, gamma-glutamyl transpeptidase; WHO, World Health Organization histological classification; TNM, tumor nodes metastasis-classification; AJCC, American Joint Committee on Cancer.

### Univariate and multivariate analysis

**Figure 2:**
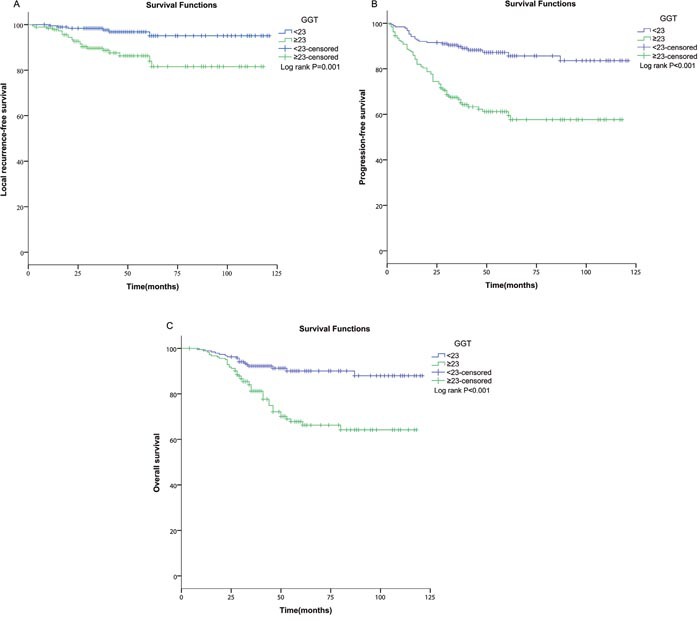
Kaplan–Meier analyses of LRFS, PFS and OS according to pretreatment serum GGT level Compared to the low subset (GGT<23U/L), elevated serum GGT level (≥23.0 U/L) had an inferior **(A)** LRFS (p=0.001), **(B)** PFS (p<0.001) and **(C)** OS (p<0.001). ROC, receiver-operating characteristic; GGT, gamma-glutamyl transpeptidase; LRFS, local relapse-free survival; PFS, progression-free survival; OS, overall survival.

Univariate analyses were performed using gender, age, tumor classification, lymph node classification, TNM stage, histology and GGT as possible variables. N-stage (p=0.044), TNM stage (p=0.023) and GGT (p=0.001 Figure 2A) were found to have relationships with inferior LRFS. Multivariate analysis confirmed that N-stage (HR, 2.549; 95% CI, 1.545-4.771; p=0.047), TNM stage (HR, 4.599; 95% CI, 1.087-19.447; p=0.038) and GGT (HR, 4.162; 95% CI, 1.690-10.25; p=0.02) were independent risk factors for LRFS. Results were showed in Table 2. Univariate survival analysis also revealed an association between worse PFS and advanced T-stage (p<0.001), advanced N-stage (p<0.001), later TNM stage (p<0.001) and GGT≥23U/L (p<0.001, Figure 2B). Multivariate analysis ensured that advanced T-stage (HR, 1.847; 95% CI, 1.106-3.084; p=0.019), advanced N-stage (HR, 1.827; 95% CI,1.126-2.966; p=0.015), later TNM stage (HR, 4.757; 95% CI, 1.316-17.201; p=0.017) and GGT≥23U/L (HR,3.119; 95% CI,1wctors for PFS (Table 3). As showed in Table 4, advanced T-stage (p<0.001), advanced N-stage (p<0.001), later TNM stage (p<0.001), and GGT≥23U/L (p<0.001, Figure 2C) were statistically significantly associated with poorer OS. Other characteristics did not show a significant correlation with prognosis. Multivariate analysis was performed according to the meaningful variables above. Moreover, advanced T-stage (HR, 1.967; 95% CI, 1.070-3.588; p=0.027), later TNM stage (HR, 4.231; 95% CI, 0.833-20.270; p=0.01) and GGT≥23U/L (HR, 2.811; 95% CI, 1.614-4.896; p=0.007) were independent prognostic factors for OS.

**Table 2:** Univariate and multivariate analysis of clinicopathological parameters for the prediction of local recurrence-free survival in patients with nasopharyngeal carcinoma (n=374)

Parameter	Univariate analysis		Multivariate analysis	
	HR(95% CI)	P-value	HR(95% CI)	P-value
**Gender**				
Female	1(Referent)			
Male	2.338(0.888-6.151)	0.085		
**Histology**				
WHO Type 2	1(Referent)			
WHO Type 3	0.976 (0.469-2.030)	0.947		
**Age at diagnosis (years)**				
<50	1(Referent)			
≥50	1.003(0.431-2.209)	0.991		
**Tumor classification**				
T1+2	1(Referent)			
T3+4	1.477 (0.703-3.102)	0.303		
**Lymph node classification**				
N0+1	1(Referent)			
N2+3	2.037(0.954-4.346)	0.044	2.549(1.545-4.771)	0.047
**TNM stage(AJCC)**				
I+II	1(Referent)			
III+IV	5.341(1.266-22.540)	0.023	4.399(1.089-19.447)	0.038
**GGT**				
<23	1(Referent)			
≥23	4.559(1.854-11.215)	0.001	4.163(1.690-10.251)	0.020

GGT, Gamma-glutamyl transpeptidase; WHO, World Health Organization histological classification; TNM, tumor nodes metastasis-classification; AJCC, American Joint Committee on Cancer; HR, hazard ratio; 95% CI, 95% confidence interval.

**Table 3:** Univariate and multivariate analysis of clinicopathological parameters for the prediction of progression-free survival in patients with nasopharyngeal carcinoma (n=374)

Parameter	Univariate analysis		Multivariate analysis	
	HR(95% CI)	P-value	HR (95% CI)	P-value
**Gender**				
Female	1(Referent)			
Male	3.272 (1.781-6.01)	0.091		
**Histology**				
WHO Type 2	1(Referent)			
WHO Type 3	0.813(0.539-1.227)	0.324		
**Age at diagnosis (years)**				
<50	1(Referent)			
≥50	1.154(0.751-1.773)	0.513		
**Tumor classification**				
T1+2	1(Referent)			
T3+4	2.876(1.814-4.558)	<0.001	1.847 (1.106-3.084)	0.019
**Lymph node classification**				
N0+1	1(Referent)			
N2+3	2.550(1.644-3.953)	<0.001	1.827 (1.126-2.966)	0.015
**TNM stage(AJCC)**				
I+II	1(Referent)			
III+IV	11.791(3.728-37.225)	<0.001	4.757(1.316-17.201)	0.017
**GGT**				
<23	1(Referent)			
≥23	3.465 (2.174-5.523)	<0.001	3.119 (1.955-4.976)	0.031

GGT, Gamma-glutamyl transpeptidase; WHO, World Health Organization histological classification; TNM, tumor nodes metastasis-classification; AJCC, American Joint Committee on Cancer; HR, hazard ratio; 95% CI, 95% confidence interval.

**Table 4:** Univariate and multivariate analysis of clinicopathological parameters for the prediction of overall survival in patients with nasopharyngeal carcinoma (n=374)

Parameter	Univariate analysis		Multivariate analysis	
	HR(95% CI)	P-value	HR (95% CI)	P-value
**Gender**				
Female	1(Referent)			
Male	2.615 (1.332-5.134)	0.073		
**Histology**				
WHO Type 2	1(Referent)			
WHO Type 3	0.787 (0.482-1.960)	0.338		
**Age at diagnosis (years)**				
<50	1(Referent)			
≥50	1.172 (0.701-1.960)	0.545		
**Tumor classification**				
T1+2	1(Referent)			
T3+4	2.289 (1.640-4.880)	<0.001	1.967(1.070-3.588)	0.027
**Lymph node classification**				
N0+1	1(Referent)			
N2+3	3.108 (1.814-5.325)	<0.001	2.249(1.243-4.070)	0.071
**TNM stage(AJCC)**				
I+II	1(Referent)			
III+IV	12.630(3.087-51.672)	<0.001	4.231(0.883-20.270)	0.01
**GGT**				
<23	1(Referent)			
≥23	3.232 (1.858-5.623)	<0.001	2.811(1.614-4.896)	0.007

GGT, Gamma-glutamyl transpeptidase; WHO, World Health Organization histological classification; TNM, tumor nodes metastasis-classification; AJCC, American Joint Committee on Cancer; HR, hazard ratio; 95% CI, 95% confidence interval.

### Subgroup analysis stratified by clinical stages

**Figure 3:**
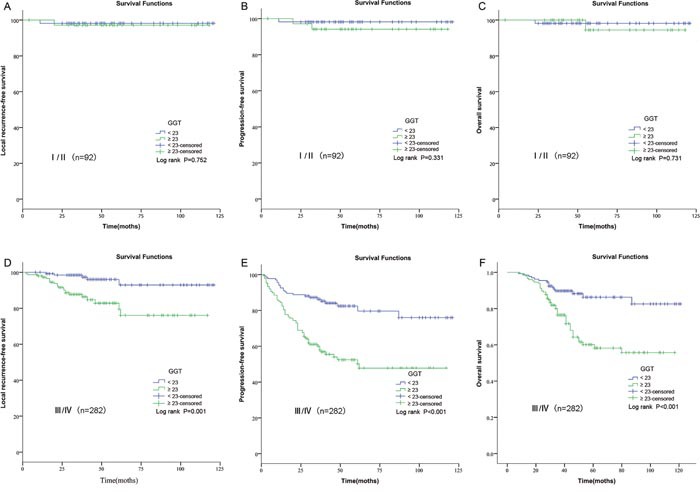
Kaplan–Meier curves for LRFS, PFS and OS rates according to pretreatment GGT in patients with different clinical stages **(A)** LRFS stratified by GGT in patients with early-stage NPC. **(B)** PFS stratified by GGT in patients with early-stage NPC. **(C)** OS stratified by GGT in patients with early-stage NPC. **(D)** LRFS stratified by GGT in patients with advanced-stage NPC. **(E)** PFS stratified by GGT in patients with advanced-stage NPC. **(F)** OS stratified by GGT in patients with advanced-stage NPC. GGT, gamma-glutamyl transpeptidase; LRFS, local relapse-free survival; PFS, progression-free survival; OS, overall survival; NPC, nasopharyngeal carcinoma.

Patients with different clinical stages were included in our study. This would cause bias, for example, the TNM stage was not balanceable in GGT groups, and the treatments were different between patients with early stage and patients with advanced stage. Thus, we further performed a subgroup analysis and evaluated the prognostic roles of GGT in NPC patients with different clinical stages. No statistical significances were observed in the early-stage subgroup (p=0.752 for LRFS; p=0.331 for DMFS; p=0.731 for OS; Figure 3A-3C). However, positive results were obtained in the advanced subgroup (p=0.001 for LRFS; p<0.001 for DMFS; p<0.001 for OS; Figure 3D-3F).

### Subgroup analysis stratified by different genders

**Figure 4:**
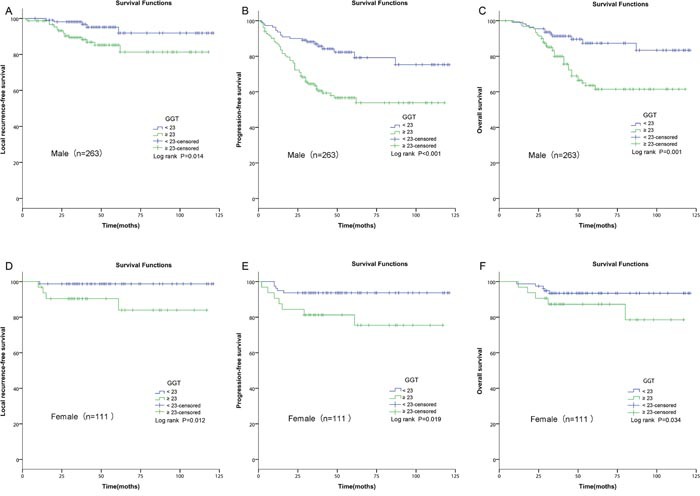
Kaplan–Meier curves for LRFS, PFS and OS rates according to pretreatment GGT in patients with different genders **(A)** LRFS stratified by GGT in male patients with NPC. **(B)** PFS stratified by GGT in male patients with NPC. **(C)** OS stratified by GGT in male patients with NPC. **(D)** LRFS stratified by GGT in female patients with NPC. **(E)** PFS stratified by GGT in female patients with NPC. **(F)** OS stratified by GGT in female patients with NPC. GGT, gamma-glutamyl transpeptidase; LRFS, local relapse-free survival; PFS, progression-free survival; OS, overall survival; NPC, nasopharyngeal carcinoma.

As shown in previous study, the normal reference range of GGT was not same [21]. In order to avoid the bias, we further performed a subgroup analysis and evaluated the prognostic roles of GGT in NPC patients with different clinical genders. GGT was identified as a positive predictor simultaneously among male and female in Figure 4.

## DISCUSSION

In recent years, improvements in diagnostic methods, radiotherapy techniques, chemotherapy regimens and novel therapies have offered vital survival benefits in locally advanced NPC [22–24]. However, more than 20% advanced NPC patients developed distant metastasis after treatment, making accurate prognostic evaluation at diagnosis extremely important for maximizing therapy benefits [25, 26]. Patients even with the same TNM stages could have dramatically different survival results [7]. Therefore, various molecular biomarkers have been studied to satisfy the demand of a more accurate prognostic system [5, 6, 27–29].

Abberant expression of GGT was found in several human tumors, including renal cell carcinoma [14], esophageal squamous cell carcinoma [15], colorectal cancer [17], and breast cancer [18], cervical cancer [19] and ovarian cancer [20]. A tight relationship was documented by many large multicenter trials between higher GGT and cancer incidence or mortality [30–33]. Cells which overexpressed GGT were shown to be more resistant to chemotherapies such as doxorubicin [34], cisplatin [11, 35, 36] and 5-fluorouracil [37] than normal cancer cells. Patients with stage IIb to IV disease then had to receive concurrent chemoradiotherapy and/or neoadjuvant or adjuvant chemotherapy with cisplatin or 5-fluorouracil [23]. Therefore, we would ascribe the weak results to the influence of GGT on medications. These studies revealed a promising prospect of GGT on the prediction of cancer and complement TNM classification. However, there were few studies focused on the relevance between GGT and NPC. Only one study identified that high GGT was not a prognostic factor for NPC [38], but it just conducted the trial with GGT level above normal and did not determined a cutoff value. Moreover, the low percentage of patients with high GGT in that study couldn't meet the need of statistical analyses. In our study, we identified a meaningful cutoff value and found that high GGT had a significant relationship with poor prognosis of NPC patients.

In this study, GGT≥23U/L was identified as a potential biomaker for LRFS, PFS and OS. However, GGT couldn't be identified as a meaningful biomarker for DMFS because of its inferior AUC and P value. Subsequently, the associations of the GGT with the clinical–pathological features of NPC were investigated. Patients classified as male, higher N-stage and advanced TNM stage were more likely to have elevated GGT. Healthy men different from women were inclined to have higher upper reference interval of GGT [21], which might explain why elevated GGT was found in men. The results were consistent with the previous studies on the associations between GGT and clinical–pathological parameters [15]. In order to avoid the deflection between different genders, Kaplan–Meier analysis was performed. GGT was identified as a positive predictor simultaneously among male and female (Figure 4). Besides, GGT was elevated in the NPC patients with higher N-stage, and lymph node status was a strong predictor for NPC patients. The impact of GGT on prognosis might result from the association between GGT and lymph node status. Univariate survival analysis and multivariate analysis confirmed that GGT and lymph node status were positive prognostic factors for NPC patients. We also found a link between elevated GGT and advanced TNM stage which was different from other studies [16, 18]. The difference might be due to tumor heterogeneity and different cut-off values. When univariate survival analysis and multivariate analysis were performed, we successfully identified that GGT was an independent prognostic factor for NPC patients. High pretreatment GGT level was correlated with poor 5-year LRFS (HR, 4.162; 95% CI, 1.690-10.251; P=0.02), PFS (HR, 3.119; 95% CI, 1.955-4.976; P=0.031) and OS (HR, 2.811; 95% CI, 1.614-4.896; P=0.007).

In our subgroup analysis, significant associations of GGT with prognosis were observed among patients with advanced stage. For the early stage group, there were no statistical significances between GGT and prognosis. Our results indicated that GGT seemed to be more effective for patients with advanced stage. Patients with NPC with early clinical stage tended to survive longer without progression. In our study, only 6 cases progressed and 2 patients died among the 92 patients with early-stage disease. The small number of outcome (progression or death) might attribute this nonsignificant association in the early stage. This subgroup analysis would make our study more intuitive and scientific.

GGT anchored in the cell membrane is a cell surface glycoprotein which can regulate the glutathione (GSH) metabolism. Protein synthesis is starving of the recovery of cysteine mediated by GGT especially in rapidly dividing neoplastic cells [39]. Meanwhile, GGT have been documented by large amount of studies to modulate the crucial redox related courses [40, 41], such as antioxidant/antitoxic defenses and cellular proliferative/apoptotic balance. The pro-oxidant reactions produced by GGT could supplement endogenous Reactive oxygen species (ROS) in cancer cells, and endogenous ROS can contribute to the ‘persistent oxidative stress’, which is described as a factor in genomic instability and carcinogenesis [42]. Furthermore, a great number of cytokines including tumor necrosis factor alpha (TNF-alpha) [43], and interferon (IFN)-alpha and -beta [44], could induce GGT mRNA expression. These results indicate a connection between inflammation and GGT expression, not just as a response to inflammation-related oxidative stress, but rather as the effect of specific inflammatory cytokines. Thus, GGT may influence the survival outcome of NPC patients by interacting with the GSH metabolism, oxidative stress and inflammation. However, the underlying mechanism still needs to be further explored *in vivo* and *in vitro*.

To the best of our knowledge, this was the first report that successfully confirmed a prognostic impact of pretreatment serum GGT in patients with NPC. This easily acquired enzyme in routine biochemical examination may complement TNM classification and allow patients to be further categorized based on the GGT, thus enable more individual treatments to be executed. Other serious diseases and medications which would affect the blood serum level of GGT were excluded. Hepatobiliary disease, concomitant malignant disease, congestive heart failure, anticonvulsants and alcohol abusewere excluded to minimize interferences. However, improper affirmation of prognostic markers is a paramount problem in current biomarker study. As GGT has been identified as a meaningful predictive biomarker for NPC patients, subsequent evaluation of its clinical benefit as a prognostic predictor in a large multi-center study needs to be carried out. Our original and objective research will offer a promising biomarker candidate for NPC and supplement the individualized treatment of NPC.

As a typical retrospective study, our study has some limitations such as lack of random assignments and its single-institution. Besides, the number of included patients in our study was limited. Moreover, not all hematologic markers were adopted in our analysis because of our inability to obtain them, such as the LDH [5], serum alkaline phosphatase [38], albumin [45], Epstein-Barr virus [7], c-reactive protein [8] etc. Despite the potential weaknesses, our results are clinically meaningful and might be a useful hypothesis for future trials.

We concluded that GGT was associated with gender, N-stage and TNM stage in patients with NPC. Pretreatment GGT was identified as a novel and independent prognostic biomarker for NPC patients. Future large randomized trials are warranted to confirm and update our results.

## MATERIALS AND METHODS

### Patient population

We collected data from 374 patients who were pathologically diagnosed with NPC and treated in the Cancer Center, Tongji Hospital between January 2005 and December 2010. This study was approved by our Ethics Committee. The inclusion criteria for patients in this study were as follows: (1) histological diagnosis with NPC; (2) having routine check up before radical treatment; (3) no radiotherapy or chemotherapy before collection of blood for GGT measurements; (4) no evidence of distant metastasis or secondary carcinoma at first diagnosis; (5) complete radical radiotherapy, with or without chemotherapy; and (6) absence of other serious diseases and medications which would affect the blood level of GGT (i.e., concomitant malignant disease, acute or chronic pancreatitis, acute or chronic hepatitis, liver cirrhosis, intra- or post-hepatic biliary obstruction, cholangitis, congestive heart failure NYHA III–IV, anticonvulsants, alcohol abuse, and so on).

### GGT measurement

As a part of clinical routine blood chemistry analysis, serum GGT was measured within 1-3 days before therapy in the morning after an overnight fast. Serum GGT concentrations were analyzed with an enzyme kinetic assay (Modular Hitachi 7600 and Hitachi 7080, Hitachi High-Technologies Corporation Tokyo, Japan). All the serum levels of GGT were detected in the same instrument by using the same reagent methodology.

### Treatment

All patients underwent a pretreatment baseline evaluation, including complete medical history, physical and neurological examinations, hematology and biochemistry profiles, MRI scan of the neck and nasopharynx, chest radiography, whole body bone scanning and abdominal sonography. Union for International Cancer Control/American Joint Committee on Cancer (UICC/AJCC) 2002 staging system was used to classify patients. Treatment plans were determined according to standard protocols depending on tumor stages and general health. According to our institutional guidelines, patients with stage I to IIa disease generally only received radical radiotherapy, whereas patients with stage IIb to IV disease received CCT and/or NACT or ACT chemotherapy. All patients received radical radiotherapy using conventional or intensity-modulated radiotherapy. The following cumulative doses were delivered to each region: nasopharyngeal region, 68–70 Gy; involved cervical node, 60-66 Gy. In addition to radiotherapy, certain patients also received CCT, NACT and ACT. The regimens of NACT included DP (docetaxel 75 mg/m^2^ IV on day 1 plus cisplatin 90 mg/m^2^ IV on day 1, repeat every 3 weeks) and PF (cisplatin 90 mg/m^2^ IV on day 1 plus 5-fluorouracil 750 mg/m^2^/d continuously IV on day 1-5, repeat every 3 weeks). CCT was performed during the period of radiotherapy (cisplatin 30 mg/m^2^ on day 1, repeat every week). For patients received ACT, the DP or PF regimens (the same as NACT) were repeated every 3 weeks for 2-4 cycles.

### Follow-up

After treatment, patients were followed-up with routine checkups in our hospital every 3 months during the first 3 years after treatment, every 6 months for the next 2 years and subsequently every year. LRFS was defined as the time from diagnosis to the date of local recurrence or the date of death or when censored at the latest date. DMFS was defined as the time from diagnosis to the date of distant metastases or the date of death or when censored at the latest date. PFS was defined as the time from diagnosis to the date of local failure/distant metastasis or the date of death or when censored at the latest date. OS was defined as the time from diagnosis to the date of death or last follow-up visit if the patients were still alive.

### Statistical analysis

ROC curve generated by MedCalc 9.6.2.0 (MedCalc Software, Mariakerke, Belgium) was used to determine the optimal cutoff value for GGT that yielded the highest combined sensitivity and specificity with respect to distinguishing 5-year survivors from non-survivors. According to the cutoff value of GGT, NPC patients were divided into high-risk group and low-risk group. Chi-square test was performed to evaluate the associations between the clinical–pathological variables and GGT. Kaplan–Meier method and log-rank test were adopted to calculate and compare the LRFS, DMFS, PFS and OS rates. Univariate and multivariate analyses were applied to determine independent factors that were significantly related to the prognosis. All statistical analyses were performed with SPSS19.0 (SPSS, Chicago, IL). A two-sided p-value less than 0.05 was considered statistically significant.

## SUPPLEMENTARY MATERIALS TABLE


