# Supplementary material for: High pretreatment serum gamma-glutamyl transpeptidase predicts an inferior outcome in nasopharyngeal carcinoma
Source: Oncotarget. 2017 Jun 28;8(40):67651–62. doi: 10.18632/oncotarget.18798 (PMC5620200; doi:10.18632/oncotarget.18798)
Supplement: Supplementary file 1 [file oncotarget-08-67651-s001.pdf]

High pretreatment serum gamma-glutamyl transpeptidase predicts an inferior outcome in nasopharyngeal carcinoma

SUPPLEMENTARY MATERIALS

Supplementary Table 1: Details about of how to select patients into the study team

Reason	The number of excluded patients
**Comorbidity**	
Hepatobiliary disease	2
Concomitant malignant disease	2
Congestive heart failure	1
**Acquired radiotherapy or chemotherapy before**	21
**Missing clinical data**	13
**Refusing any treatment**	3
**Distant metastasis at first diagnosis**	5
**Loss of follow-up**	91
**Alcohol abuse**	7
